# The Evaluation of saccharose replacing by adding stevioside‐maltodextrin mixture on the physicochemical and sensory properties of Naanberenji (an Iranian confectionary)

**DOI:** 10.1002/fsn3.463

**Published:** 2017-04-24

**Authors:** Amirpouya Ghandehari Yazdi, Mohammad Hojjatoleslamy, Javad Keramat, Mahshid Jahadi, Elahe Amani

**Affiliations:** ^1^ Food Science and Technology Department of Food Science and Technology Shahrekord Branch Islamic Azad University Shahrekord Iran; ^2^ Food Science and Technology Department of Food Science and Technology Faculty of Agriculture Isfahan University Of Technology Isfahan Iran; ^3^ Food Science and Technology Department of Food Science and Technology College of Agriculture Isfahan (Khorasgan) Branch Islamic Azad University Isfahan Iran; ^4^ Food Science and Technology Department of Food Science and Technology College of Agriculture Shiraz University Shiraz Iran

**Keywords:** Healthy Diet, Naanberenji, Stevioside‐maltodextrin, Sucrose

## Abstract

Stevia is a natural, non‐nutritive sweetener can replace sugar in your diet to control diabetes and aid in weight loss. Naanberenji, an Iranian traditional cookie, is a well‐reputed confectionary containing high sucrose and calorie value, for this reason its consumption in healthy diet has been restricted. In this study, the effect of sucrose replacement by stevioside‐maltodextrin mixture on physicochemical properties of Naanberenji was evaluated in four replacing levels including 25%, 50%, 75%, and 100%. Results of texture evaluation revealed that hardness increases due to adding stevioside‐maltodextrin (*p* < .05). Color analysis also showed that browning index by adding more mixture reduced. Moreover, the results of sensory analysis showed that treatment containing 25% stevioside‐maltodextrin was the most similar sample to control and 23.06% reduction in sugar consumption achieved in this level of replacement. Therefore, its physicochemical properties (peroxide, acidity, chemical compounds, and hardness) were measured. According to the results, calorie amount decreased by 7.27 %. While there was no significant difference in the acidity of the aforesaid sample, peroxide results exhibited significant differences. Analysis of Farinograph plot revealed more water binding in comparison to control sample.

## 1 INTRODUCTION

Changes in lifestyle, reduction in human being's mobility, incidence of obesity, hypertension, and heart disease necessitate of low calorie formulas. Therefore, to people suffer from diabetes, obesity, children, and pregnant women, consummation less sucrose is recommended (Glicksman & Farkas, 1966). Mainly because sucrose intake has shown indications of being a major contributor to adolescent obesity (Striegel‐Moore et al., 2006). Therefore, many researchers and producers have attempted to find sucrose alternatives that have the same browning, structural, and sweetening capabilities.

**Figure 1:**
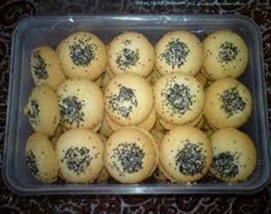
Rice bread, Iranian traditional confectionary

**Figure 2:**
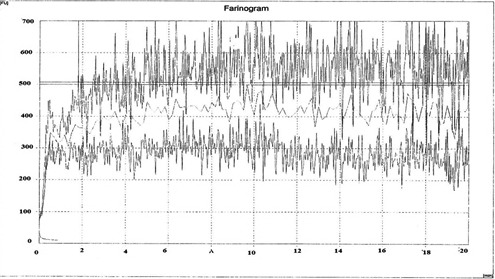
Rice flour farinograph

Non‐nutritive sweeteners provide unique benefits and properties that could help to control health and prevention of diabetes. For instance, consumption of these sweeteners may enhance weight loss (Raben, Vasilaras, Moller, & Astrup, 2002), reduced energy intake (Wiebe et al., 2011), and reduced BMI (Wiebe et al., 2011). Furthermore, the annual growth, competition entry, and product development that this market has experienced in the latter part of the twentieth century (Prance, 2007) is also clear evidence of increasing demand for them. Naanberenji which is a well‐accepted Iranian traditional cookie compose of rice flour, sucrose, egg, and oil in two small and big sizes (Figure 1) (Martinez et al., 2012a, 2012b). Recently, stevia and its sweetener called stevioside have been taken into account as an appropriate replacer for sucrose. This plant is founds in the form of a short herby plant near Paraguay Brazil border (Carakaostas, Curry, Boileau, & Brusick, 2008). Researches have discovered that diterpene glycosides are responsible for stevia sweetness (Hamzeloei et al., 2004) which estimated to be 200–300 times as much as sucrose (Mogra & Dashora, 2009). Findings exhibit that stevia can be applied in treating diabetes, exhaustion, cancer, hypertension, and it prevents mouth microorganism growth (Mogra & Dashora, 2009; Curry & Roberts, 2008; Ghosh, Subudhi, & Nayak, 2008;  Fatemi, 2007). Figure 2 depicts the chemical structure of stevioside. In which nonsugar part is diterpene called steviol containing a hydroxyl group in its 4th carbone (Keramat, 2007; Ahmed, Hossain, Islam, Kumar Saha, & Mandal, 2011). In recent decades, stevioside has been consumed in beverages and confectioneries in China, USA, and Japan (Koyama et al., 2003). In addition to a dietary sugar, filling agents such as alcoholic sugars, maltodextrin, and nondigestible carbohydrate like cellulose is required as well to produce sugar‐free confectioneries (Bullock, Handu, Segall, & Wasserman, 1992). Nowadays, different modified starch compounds are widely applied in food industries. Maltodextrin, one of the modified starch types (Equivalent Dextrose <20) is composed of a mixture compounds with molecular weight between poly and oligo saccharides in white powder and concentrated syrup forms (Sadeghi, Shahidi, Mortazavi, Mahalati, & Beheshti, 2007). In comparison to crude starch, maltodextrin enjoys more water solubility, being cheaper than most edible hydrocolloids with no flavor and color; besides, it can be taken into account as a gelling agent, a consistency creating agent, a viscosity increasing agent, it increases dry solid, resists high temperatures, prevents crystallization and, controls freezing temperature (Chronakis, 1998; Docik‐Baucal, Dokic, & Jakovljevic, 2004). In recent years, studies have focused on the production of cereal low calorie products. Study of mutual affects of stevioside, liquid sorbitol, xanthan gum, and emulsifier revealed that viscosity is reduced in sucrose replacing sorbitol, whereas viscosity is increased by adding gum; moreover, in another study, the effect of sucralose and polydextrose on rheological properties, texture, structure, color, and appearance of Muffin exhibited no acceptability of samples at all sucrose replacement (Manisha, Soumya, & Indrani, 2012). Adding erythrol and xanthan mixture along with leavening agent enhances the product volume and reduces hardness (Martinez et al., 2012a, 2012b).

Applying stevia as a sweetener on biscuit exhibited the reduction in peroxide value; in fact, by increasing the amount of sweetener, peroxide value decreased (Hamzeloei et al., 2004), moreover, in 80% sucralose‐dextrin replacement in chiffon cake, hardness was increased and desirability was reduced (Lin & Lee, 2005). This article aims at the investigation of affections of stevioside‐maltodextrin mixture on textural and sensory properties of the Naanberenji by partially or completely replacing of its sugar.

**Figure 3:**
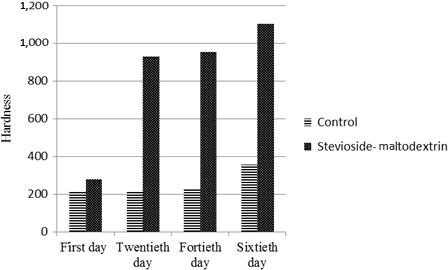
The affection of sucrose replacement with stevioside‐maltodextrin mixture on Naanberenji hardness during shelf life

## 2 MATERIAL AND METHODS

### 2.1 Raw material

Consumed ingredients include rice flour (North, Iran), sugar (Chaharmahal Suagar Co, Shahrekord, Iran), rosewater, oil (Varamin, Tehran, Iran), and maltodextrin (Dextrose Equivalent < 20) from Poran powder, Isfahan, Iran, and stevioside from Beno Co, Germany purchased from market.

### 2.2 Preparing Naanberenji samples:

Firstly, oil, sugar, and egg yolk were mixed. White yolk was individually blended after that added to the mixture. Rosewater and extra sugar were prepared and completely shaken, then, rice flour was added and shaking continued to reach a uniform and slightly loose dough. Prepared dough was placed in a nylon bag for 24 h. Eventually it was developed to 0.5 cm thickness and was cut with round molds. It was placed in an oven at 300°C for 15–20 min, after cooling, all samples were placed in packaging containers.

To reduce the amount of sugar, four levels of stevioside‐maltodextrin mixture, 25%, 50%, 75%, and 100% added so that four treatments and control were experimented.

### 2.3 Physiochemical properties of rice flour

Experimental test approved by AACC including, moisture (44–16), ash (08–01), protein (46–12), fat (30–20), and falling number (56081) were performed on consumed rice flour (AACC, 2000).

### 2.4 Naanberenji experiments

#### 2.4.1 Sensory evaluation

Scoring the intensity of a feature was employed for sensory evaluation where five trained panelists in the field of taste and texture of naanberenji assessed eight treatments containing stevioside‐maltodextrin and control with three random digits as code. Distilled water was used for washing mouth. All the samples were analyzed based on four characteristics including aftertaste (1 = without aftertaste, 5 =  with high aftertaste), texture (1 =  extremely soft, 5 =  extremely hard), sweetness (1 =  without sweet taste, 5 =  extremely sweet), and overall desirability (1 =  extremely brackish, 5 =  extremely tasty).

#### 2.4.2 Investigation of naanberenji rheological properties

In fact, the required force to penetrate a bar in inner parts of foodstuff is a scale to assess the amount of hardness (Bourne, 2002). Brookfield Engineering middle base, CT3‐4500, USA, were used for performing the punching test (in three replicates, blade race and penetration thickness were considered 0.5 mm/s and 10 mm, respectively. Selected probe was Probe TA39 with following characteristics: rod shape, stainless steel, length 20 mm, diameter 5 mm, and weight 5 g (Haj Mohammadi, Keramat, Hojatoleslami & Maulavi, 2013).

#### 2.4.3 Color analysis

To analyze color, providing uniform and constant condition is very important; therefore, the samples were placed in a box with an internal white wall. Two 18w lamps with 30° as irradiance angel supplied the requested light, then pictures of samples was taken by canon camera, model IXUS130. Image processing software was used to convert RGB to *L, *a, *b. Equation (1) and Equation (2) were employed for the measurement of color changes and browning index (Saricoban & Yilmaz, 2010).

(1) $$\Delta E=\sqrt{{\left(L^{\circ }-L\right)}^{2}+(a^{\circ }-a{)}^{2}+{\left(b^{\circ }-b\right)}^{2}}$$

(2) $$B=\frac{100\times (X-0/31)}{0/17}$$

In which **X** is; $X=\frac{\left(a+1/75\times L\right)}{(5/645\times L+a-3/012\times b}$

The best sample was selected after these experiments, then chemical experiments, calorie amount, stability, and rheological properties were performed on it and control sample.

#### 2.4.4 Investigation of crude calorie

Naanberenji calorie was assessed by a calorimeter bomb (England) on the control sample and the best treatment containing stevioside‐maltodextrin mixture on three replicates. Samples were homogenized, and placed in an oven for 24 h (to reach the energy amount of 100 dry solid), then 0.5 g of this homogenous mixture was placed in a small chamber, after that oxygen valve was opened to combust the sample. The produced heat was transfered to galvanometer. Equation (3) was used to measure the energy resulting.

(3) $$E=\frac{466.54*(D-0.1)}{c}e$$

In which c is the sample weight, D is the galvanometer number, 0.1 is energy produced from rope burning, and 466.54 is equivalent to energy resulting from benzoic acid burning.

### 2.5 Stability and chemical experiments

Experiments including, ash, fat, protein, moisture, peroxide index, and acidity during storage time (2 month, every 30 days) were accomplished on the control and the sample containing sucralose‐maltodextrin.

### 2.6 Rheological experiments

Punch test was performed every 20 days on two aforesaid treatments. To evaluate the effect of stevioside‐maltodextrin mixture on rheological properties of the dough, all the compounds except rice flour were removed, then to control sample rice flour, sucrose was added and for treatment containing stevioside‐maltodextrin rice flour, sucralose and maltodextrin were mixed and examined via farinograph experiment using statistical analysis method. SAS v.9 and Excle 2007 software was used to conduct ANOVA test in a completely random design and comparing LSD average for four Naanberenji samples.

**Table 1:** Low calorie Naanberenji formulas containing stevioside‐maltodextrin

Ingredients per 100 gr of flour	25% Stevioside‐Maltodextrin	50% Stevioside‐Maltodextrin	75% Stevioside‐Maltodextrin	100% Stevioside‐Maltodextrin
Stevioside	0.43	0.87	1.31	1.75
Maltodextrin	87.07	174.10	261.19	348.25
Sucrose	262.50	175	87.50	–
Egge	100	100	100	100
Rosewater	170	170	170	170
Oil	500	500	500	500
Rice flour	1000	1000	1000	1000

## 3 RESULTS AND DISCUSSION

### 3.1 Physiochemical properties of rice flour

Physiochemical properties of rice flour showed in Table 1. Rice flour is composed of 10.16% protein, 0.76% ash, 0.8% fat, 6.2% moisture, nonacid soluble ash 0.04%, and falling number is 999s due to high amount of rice flour creating a hard gel so that viscometer shaker can barely break it. Consumed flour contains 54.2% water holding capacity, 20 m as developing time, and 18.1 m as stationary time (Fig. 2).

Results of texture evaluation are completely verified by sensory evaluation results (Table 2). There were no significant differences in sweetness intensity (up to 75%) except samples containing 100% stevioside that had the least sweetness. There are some problems in using of stevioside (Keramat, 2007), for example, bitter aftertaste of stevioside, delay in stevioside releasing flavor due to the presence of some essential oils, tanan, and flavonoids along with rebadioside A (Mogra & Dashora, 2009). Presence of flavoring compounds like rosewater probably covers stevioside aftertaste, and replacement in level of 25% resulted in reduction in overall desirability (*p* < .05). These results are in agreement with previous studies (Martinez et al., 2012a, 2012b; Lin & Lee, 2005).

**Table 2:** The investigation of sucrose replacing with stevioside‐maltodextrin mixture on sensorial properties of rice bread

Treatments	Texture	Sweetness	Aftertaste	Overall liking
Control	5.0 ± 0.7^a^	3.2 ± 0.4^a^	1.0 ± 0.0^a^	7.0 ± 0.0^a^
25%St‐MD^a^	5.6 ± 0.5^b^	3.2 ± 0.4^a^	1.0 ± 0.0^a^	7.2 ± 0.8^a^
50%St‐MD	7.0 ± 0.7^c^	3.0 ± 0.7^a^	1.0 ± 0.0^a^	6.0 ± 0.7^a^
75%St‐MD	7.0 ± 0.0^c^	2.6 ± 0.5^ab^	1.0 ± 0.0^a^	5.6 ± 0.5^b^
100%St‐MD	7.0 ± 0.7^c^	2.2 ± 0.4^b^	1.8 ± 0.4^b^	4.8 ± 0.4^c^

Those numbers with at least one common letter have no significant difference (*p* < .05).

a St‐MD: Stevioside‐maltodextrin.

### 3.2 Analysis of penetration test related to treatment containing stevioside‐maltodextrin and control

Penetration test results showed in Table 3 revealed a significant increase in hardness (*p* ≤ .05), in which the least and the most required forces were related to control and treatment containing 100%, respectively. In fact, maltodextrin, which has a high molecular weight compound, is composed of Amylose and Amylopectin (Keramat, 2007) (with more amounts and ability to water uptake), so with more replacing amount, viscosity more increased; therefore, hardness also increased. Moreover, according to previous researches in sucrose replacement with alcoholic sugar, aspartame, rrythritol, oligofructose in sponge cake, and sucralose‐dextrin mixture in Chiffon cake, the increasing in hardness has also been reported (Lin & Lee, 2005; Peighambardoost et al., 2011).

**Table 3:** The assessment of sucrose replacing with stevioside‐mltodextrin on rheological properties of rice bread (Penetration test)

Treatments	Force
Control	213.66 ± 3.51^a^
25%St‐MD	276.506.24^b^
50%St‐MD	968.16 ± 18.5^c^
75%St‐MD	1811.33 ± 12.5^d^
100%St‐MD	2647.33 ± 9.00^e^

Those numbers with common letters have no significant difference (*p* < .05).

### 3.3 Color analysis of treatment containing stevioside‐maltodextrin and control

As depicted in Table 4, no significant difference was observed among treatments (*p* ≥ .05). While the most amount of reddish, yellowish, color changes, and browning index were related to control sample, the most L* was related to treatment containing 100% stevioside‐maltodextrin. Browning reaction, Maillard and caramelization occur slightly in the presence of Maltodextrin as a result of its low external reducing groups, causing significant differences in yellowish, browning index, color, and lightness changes to arise (*p* < .05) (Sadeghi et al., 2007).

**Table 4:** The assessment of sucrose replacing with stevioside‐maltodextrin on color and rheological properties of rice bread

Treatments	L*	a*	b*	∆E	BI	Hardness
Control	83.86 ± 2.00^a^	1.10 ± 1.00^a^	36.48 ± 2.01^a^	–	56.21 ± 2.96^a^	213.66 ± 3.51^a^
25%St‐MD	84.42 ± 2.08^a^	0.92 ± 0.45^a^	36.30 ± 1.52^a^	4.30 ± 2.10^a^	55.30 ± 2.21^a^	276.506.24^b^
50%St‐MD	86.03 ± 0.88^a^	0.89 ± 0.33^a^	35.32 ± 1.80^a^	4.63 ± 1.60^a^	52.01 ± 3.30^ab^	968.16 ± 18.5^c^
75%St‐MD	86.85 ± 2.06^b^	0.70 ± 1.22^a^	33.43 ± 2.20^b^	5.57 ± 2.74^b^	47.80 ± 3.20^b^	1811.33 ± 12.5^d^
100%St‐MD	87.46 ± 0.35^b^	0.64 ± 0.20^a^	27.64 ± 2.71^c^	9.71 ± 2.72^b^	37.73 ± 4.52^c^	2647.33 ± 9.00^e^

Those numbers with common letters have no significant difference (*p* < .05).

Water activity reduction observed in increasing the amount of dry solid and decreasing Millard. Indeed, Millard participants are immobilized in low moisture. Regarding to this point control sample contains sucrose, has the most browning index. Indeed, sucrose decomposes to fructose and glucose through heating and they participate in Millard reaction, the control sample showed the highest browning index. As results of heating process Glucosan and Levolosan, inverts sugars are formed and finally Hydroxymethylfurfural is created (Keramat, 2007).

### 3.4 Results related to shelf life period

#### 3.4.1 Texture investigation

**Figure 4:**
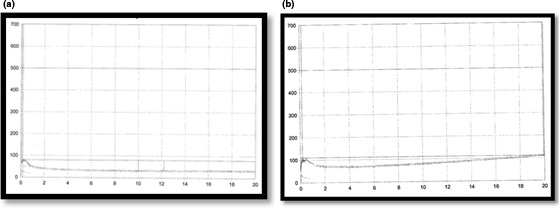
(a). Farinograph of treatment containing stevioside‐maltodextrin, (b). Farinograph of control

Figure 3 depicts that during shelf life (2 months), texture of treatment containing 25% stevioside‐maltodextrin was significantly harder than control. It is thought that increasing of water uptake (which were 35.30 and 34.50 for treatment containing stevioside‐maltodextrin mixture and control, respectively (Fig. 3) results in inversing of dough viscosity. Results coming from farinograph also verify the hardness of treatment containing 25% stevioside‐maltodextrin (Fig. 4).

#### 3.4.2 Investigation of acidity and peroxide

Results in Table 5 showed that the most peroxide index was related to the control sample and was significantly reduced by replacing sucrose by 25%. These results are in agreement with the findings of the same replacement in biscuits in which peroxide index was remarkably reduced (Hamzeloei et al., 2004).

Stevia antioxidant activity has been proved in many researches such as adding stevia to cocoa milk where the most peroxide value was observed in 100% level of stevia (Mohammadi, Delshadian, Langroudi, Homayouni Rad, & Mortazavian, 2012). Methanol extract of stevia, which contains flavonoids, alkaloids, xanthphyls, and hydroxyl cynamic acids exhibits a noticeable antioxidant activity (Carino et al., 2007). However, according to the results sucrose replacing with stevioside‐maltodextrin showed different effect, it is thought that maltodextrin increases viscosity by water uptake and this increasing leads to oil emulsification, therefore reduces antioxidant activity in sucrose replaced samples. As depicted in Table 5, the amounts of peroxide and acidity have increased during storage (*p* < .05).

**Table 5:** The investigation of sucrose replacing (25%) with stevioside‐maltodextrin mixture on acidity amount (oleic acid/100 g sample) and peroxide value (mg of oxygen/mg of oil) of naanberenji treatments

Treatments	First day	Thirtieth day	Sixtieth day
Proxid			
Control	2.91 ± 0.02^Aa^	3.72 ± 0.07^Bb^	4.64 ± 0.20^Ca^
25%St‐MD	2.74 ± 0.04^Ab^	3.21 ± 0.14^Bb^	3.97 ± 0.05^Cb^
Acidity			
Control	0.25 ± 0.02^Aa^	0.34 ± 0.02^Aa^	0.45 ± 0.02^Ba^
25%ST‐MD	0.21 ± 0.00^Aa^	0.27 ± 0.04^ABa^	0.35 ± 0.05^Bb^

Letters A,B ….in rows and a,b,….in columns exhibit the significant differences (*p* < .05).

The results of acidity has been summarized in Table 5. According to the results, acidity increased during storage. Moreover, no significant differences were observed among control and dietary treatment including stevioside‐maltodextrin mixture on the 1st, 30th, and 60th day of shelf life.

### 3.5 Evaluation of crude calorie amount in control and treatment containing 25% stevioside‐maltodextrin

Regarding the point that the target of this study was to produce low calorie naanberenji, hence the assessment of calorie reduction seems to be necessitated. Results showed that in comparison to control, by 25% replacement, calorie has decreased 7.27%. It is noted that both sucrose and maltodextrin contain 4 kcal/g (Farzanmehr, Abbasi, & Sahari, 2007).

Due to maltodextrin is polymeric component and has high molecular weight less be absorbed so that lead to reduction in calorie consumption. Although, stevioside is a free calorie sweetener, it has no important role in calorie reduction. These results are in agreement with results obtained by Bramlett, Harrison, McKemie, & Swanson, (2012). They found that sucrose replacing with sucralose‐isomalt mixture in chocolate cookie resulted in a 5%–8% calorie reduction. Swanson, Mckemite, Savage, & Zhuang, (2009) also reported that by replacing sucrose (35%) with sucralose and sucralose‐maltodextrin in cookie produced from oat, the calorie amount was reduced to 6%–7%.

### 3.6 Chemical properties of control and treatment containing 25% stevioside‐maltodextrin

Sugar evaluation is one of the important experiment in this study. According with obtained results in Table 6, sucrose replacement by stevioside‐maltodextrin mixture showed a 23.06% reduction in sugar content at level of 25% of replacement.

**Table 6:** The investigation of sucrose replacement affection by stevioside‐maltodextrin on the chemical contents of rice bread

gr/100 gr	25%St‐MD	Control
Moisture	4.18	4.01
Ash	0.61	0.56
Fat	25.4	27.4
Protein	5.86	5.85
Sugar	13.44	17.47

## 4 CONCLUSION

Results revealed, it is impossible to replace sucrose by stevioside‐maltodextrin mixture in 100% level. The most acceptable level of saccharose replacing by stevioside in formulation of Naanberenji was 25%. The main important parameter caused replacing in high level showed reverse effect is hardness that lead to reduction in desirability.

## CONFLICT OF INTEREST

None declared.
